# Effect of the intravascular low energy laser illumination during percutaneous coronary intervention on the inflammatory process in vascular wall

**DOI:** 10.1007/s10103-012-1142-z

**Published:** 2012-06-26

**Authors:** Arkadiusz Derkacz, Marcin Protasiewicz, Rafał Poręba, Adrian Doroszko, Ryszard Andrzejak

**Affiliations:** 1 Department of Internal Medicine, Occupational Diseases and Hypertension, Wroclaw Medical University, Borowska 213 Street, 50-556 Wroclaw, Poland; 2 Department of Cardiology, Wroclaw Medical University, Borowska 213 Street, 50-556 Wroclaw, Poland

**Keywords:** Low-energy laser irradiation, Percutaneous coronary intervention, Restenosis, Interleukin 1β, Interleukin 6, Interleukin 10

## Abstract

The angioplasty procedure is associated with a release of numerous factors triggering the local inflammatory reaction in vascular wall and leading thus to the restenosis. In this study, we hypothesize that the low-energy laser irradiation may exert beneficial effect by limiting this process. A group of 101 subjects (75 men and 26 women, mean age: 59.1 ± 10.3) treated with percutaneous coronary intervention (PCI), were recruited to this study. While 52 patients (40 men and 12 women) were subjected to the intravascular low-energy laser irradiation (*λ* = 808 nm) of dilated lesion during the PCI, the remaining patients (35 men and 14 women) constituted the control group. The levels of interleukin 1β, 6 and 10 (IL 1β, IL 6 and IL 10) were measured immediately before the procedure, and then at the 6th, 12th hour as well as after 1 month following the PCI. Significantly lower levels of IL 1β and IL 6 in the irradiated group during each analysis after the procedure were observed. Moreover, significantly lower IL 10 level in irradiated group within 6 and 12 hours after PCI was observed. Irradiation of the lesion with low-energy laser radiation during the PCI procedure results in a decrease in the levels of pro-inflammatory IL 1β and IL 6 as well as in an increase in the levels of anti-inflammatory IL 10, which may result in decreased risk for restenosis.

## Introduction

Percutaneous coronary intervention (PCI) is a commonly used procedure for treatment of coronary artery disease and is based on mechanical dilatation of atherosclerotic lesions. As a result, numerous factors are being released including cytokines which can increase the incidence of repeated occurrence of stenosis in the dilated segment (restenosis) [1, 2]. The activity of local inflammation is modified by interleukins.

The low-energy laser radiation may limit the local inflammatory reaction. Small number of clinical studies point at beneficial effect in preventing restenosis [3–5].

The aim of the study was to determine the effect of intravascular low-energy laser irradiation used during PCI on the activity of inflammatory process assessed by the serum levels of interleukins.

## Material and methods

### Material

All experiments were conducted and approved in accordance with the guidelines of the Bioethics Committee at the Wroclaw Medical University. The group of 101 patients with stable coronary artery disease (26 women and 75 men, mean age: 59.1 ± 10.5 years), who underwent PCI were investigated. Only subjects treated successfully (residual stenosis of up to 30 % in case of balloon angioplasty, and up to 10 % in case of stent placement) were enrolled to the study. In 29 patients, classical balloon angioplasty was performed, and in 72 patients, vascular stent was additionally implanted. All procedures were performed from the femoral artery puncture according to the Judkins technique. Patients were randomized to each group, by means of “coin flipping”. Randomization was performed by an independent physician following patients’ informed consent and the enrolment to the study. Patients were blinded to the selected method of treatment. In 52 subjects (40 men and 12 women), additional radiation of the dilatated lesion was performed (laser group). Remaining 49 subjects (35 men and 14 women) underwent classical angioplasty procedure and constitute the control group. After the procedure, all patients were given acetylsalicylic acid, clopidogrel, simvastatin and, when possible, the angiotensin-converting enzyme inhibitor and β-adrenolytic agent.

All patients had de novo stable coronary lesions of type A or B (according to the American College of Cardiology/American Heart Association classification). Subjects with diameter of coronary artery in the lesion place (reference diameter) smaller than 2.5 mm, with implanted antimitotic drug-eluting stents (DES), vulnerable plaques, ostial lesions, major calcifications, bifurcations and lesions of type C were excluded from the study. Patients with concomitant chronic disorders (such as diabetes mellitus, neoplasms, chronic inflammatory diseases) were also not included. Control coronary angiography was planned 6 months after PCI.

No significant differences in cardiovascular risk factors, such as age, smoking habit, presence of arterial hypertension, lipid profile and gender between the two analyzed groups were observed (Table 1).

**Table 1:** Demographic, clinical and procedural characteristics of analyzed groups

	Laser-treated group	Control group	*p* Values
Total number of patients	52	49	NS
Women	12	14	NS
Men	40	35	NS
Age for the total group [years]	57.3 ± 11.3	60.7 ± 9.5	NS
Age of women [years]	64.1 ± 12.1	63.5 ± 9.4	NS
Age of men [years]	55.2 ± 10.2	57.9 ± 9.7	NS
Coronary artery disease			
CCS I	12	6	NS
CCS II	21	22	NS
CCS III	12	14	NS
CCS IV	7	7	NS
Arterial hypertension	24	30	NS
Tobacco smoking	34	31	NS
Total cholesterol [mg/dl]	199.9 ± 52.0	198.3 ± 45.4	NS
LDL cholesterol [mg/dl]	123.4 ± 43.5	123.7 ± 38.6	NS
HDL cholesterol [mg/dl]	46.4 ± 9.7	42.7 ± 8.1	NS
Triglycerides [mg/dl]	151.5 ± 87.2	156.4 ± 83.6	NS
Dilated artery			
LAD	29	23	NS
RCA	12	13	NS
Cx/OM	11	13	NS
Lesion supplied with balloons			
LAD	7	3	NS
RCA	4	6	NS
Cx/OM	3	6	NS
Lesion supplied with stents			
LAD	22	20	NS
RCA	9	7	NS
Cx/OM	7	7	NS
Reference diameter of dilated artery [mm]	3.20 ± 0.44	3.11 ± 0.43	NS
Lesion length [mm]	13.6 ± 5.0	12.0 ± 3.1	NS
MLD before angioblasty	0.83 ± 0.45	0.85 ± 0.42	NS
MLD after angioplasty	2.71 ± 0.38	2.52 ± 0.37	NS
Myocardial infarction in the past, total^a^)	32	29	NS
-/- in region supplied by dilated artery	29	26	NS
-/- in another region	6	3	NS

CCS *Canadian Cardiovascular Society*, *MLD* minimal lumen diameter

^a^Number of subjects who experienced myocardial infarction in the past (in group I, every of three persons experienced two infarctions; in group II, one person experienced two infarctions). Evaluation whether infarction developed in the area supplied by the dilated artery, in some cases, may be only tentative

### Stenosis measurement

The assessment of coronary stenosis before and after 6 months following angiography was performed using quantitative coronary arteriography (QCA). This computer method gives objective data, independently from a physician evaluation. Restenosis was defined as at least 50 % stenosis in the control angiography. This value is commonly accepted as the cutoff point for the restenosis [6]. In the 6-month follow-up, the late loss of the vascular lumen (LLL) and late lumen loss index (LLL_index_) were also analyzed [7].

### Laser irradiation

Illumination of the dilated lesion was performed once, immediately after successful coronary angioplasty. We used an individual technique, based on the optical fiber capable of emitting surrounding radiation, mounted in a special balloon catheter, which provided total vascular wall radiation. During the study, we used the radiation with wavelength *λ* = 808 nm, the irradiance was 100 mW/cm^2^, and energy equalling 9 J/cm^2^ was obtained from a semiconductor laser, where the diode was optically connected (“pigtailed”) with optical fiber. This method was already presented in other papers [8–10].

### Measurement of serum levels of interleukin 1β, 6 and 10

Serum levels of interleukin 1β, 6 and 10 (IL 1β, IL 6 and IL 10) were measured using an enzyme-linked immunosorbent assay (ELISA) method (R&D Systems, UK). All assays were performed before PCI and then at 6th, 12th hour after the procedure, and during control examination 1 month after the procedure.

### Statistical analysis

Statistical analysis was performed by “Statistica PL 6.0” package (StatSoft, Poland). Data is expressed as mean ± SD. The distribution of variables was verified with Shapiro–Wilk test. In case of independent quantitative variables with normal distribution, Student’s *t*-test or ANOVA analysis were used, as appropriate. For nonparametric analyses *U* Mann–Whitney test or nonparametric Kruskal–Wallis ANOVA test were used, whereas in case of dependent quantitative variables, Wilcoxon-matched pairs test or nonparametric Friedman ANOVA variance analysis were used. Statistically significant differences were marked with Newman–Keuls post hoc test. The level of *p* < 0.05 was considered as statistically significant. Prism 5.0 Software by GraphPad was used for graphical presenting the data

## Results

### Clinical outcome

After 1 year of follow-up, we have reported one death of a patient from the control group, which was due to the myocardial infarction from another area than the dilatated artery. Control coronary angiography was performed in 40 cases in laser group and 37 subjects from the control group. In laser subjects who underwent control angiography, the restenosis was observed in six cases (15.0 %), and in the control group, in 12 cases (32.4 %). This difference was statistically insignificant. Nevertheless, we have observed a significantly smaller average stenosis in the laser-treated group (32.0 % vs. 43.5 % of the lumen, *p* < 0.05) which resulted from smaller average degree of stenosis in patients with diagnosed restenosis (59.1 % vs. 78.8 %, *p* < 0.01). In the group without restenosis, these differences were statistically insignificant. In the laser irradiation group, the control coronary arteriography also showed greater mean diameter of the vessel lumen (2.18 ± 0.70 mm vs. 1.76 ± 0.74 mm; *p* < 0.05) and smaller mean LLL (0.53 ± 0.68 mm vs. 0,76 ± 0.76 mm; *p* < 0.01) and LLL_index_ (0.28 ± 39 vs. 0.46 ±43; *p* < 0.005). There were no significant differences between mentioned above parameters dependent on the procedure (balloon angioplasty alone vs. angioplasty with stent placement). The results were shown in our previous paper [5].

### Serum interleukins’ levels

**Fig. 1:**
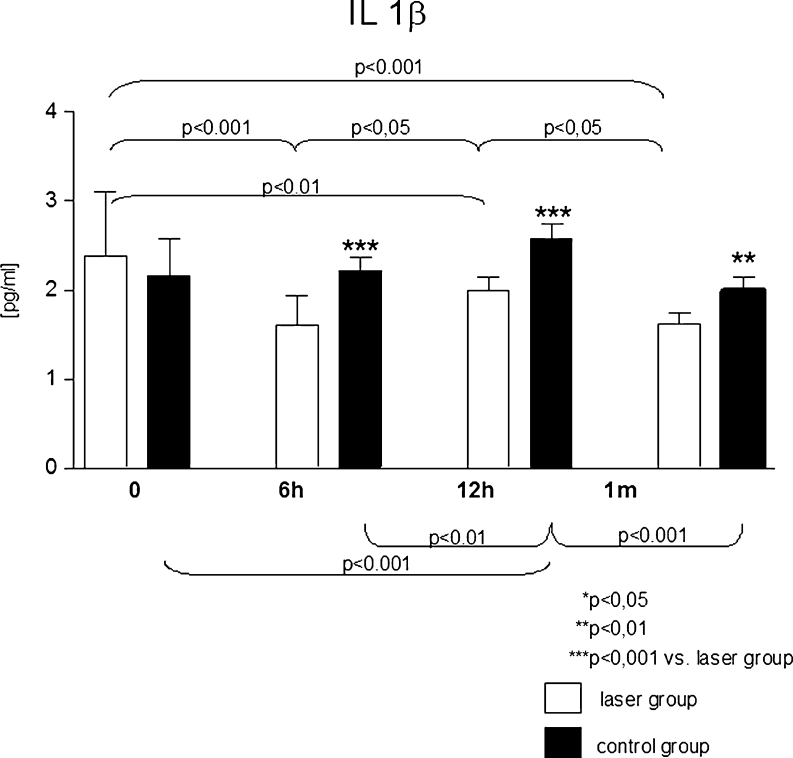
Serum interleukin 1β levels [pg/ml] in analysed groups at particular steps of the study protocol

Serum levels of IL 1β, IL 6 and IL10 in both groups were similar before the procedure. In all measurements after PCI, the levels of IL 1β were significantly higher in control group as compared to laser group. In the laser-treated group, following the PCI procedure, a decrease of IL 1β levels was observed, whereas in the control group, there was an increasing trend (Fig. 1).

**Fig. 2:**
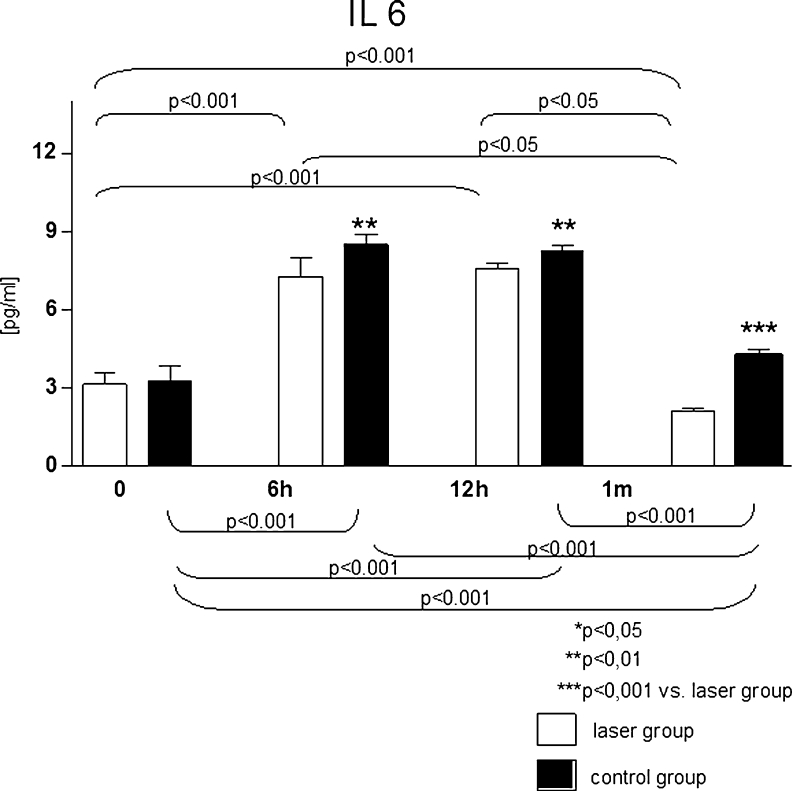
Serum interleukin 6 levels [pg/ml] in analysed groups at particular steps of the study protocol

The PCI was associated with temporary increase of IL 6 serum levels (both groups), however, after the procedure IL 6 levels were significantly higher in control group. Differences in levels of IL 6 at particular steps of experimental protocol are shown in Fig. 2.

**Fig. 3:**
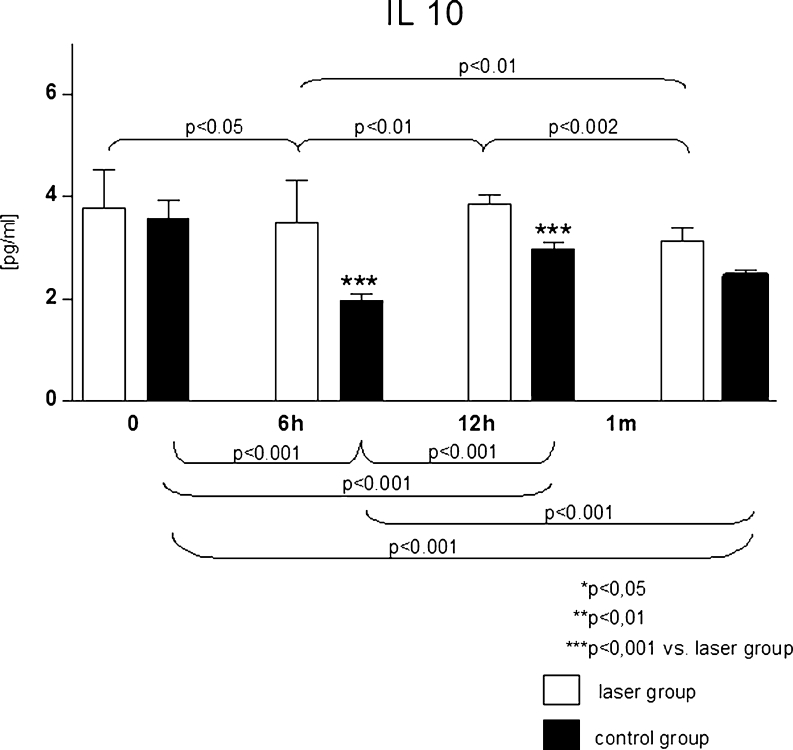
Serum interleukin 10 levels [pg/ml] in analysed groups at particular steps of the study protocol

At 6th and 12th hour following the PCI procedure, significantly higher concentration of IL 10 was observed in laser group, as compared to the control. Changes in the serum IL 10 levels at particular steps of the study protocol were also statistically significant, as presented in Fig. 3.

There were no differences in serum interleukin levels independently on the revascularization procedure (PCI vs. PCI with stent placement) in both subgroups, which was due to small number of patients enrolled to each group.

## Discussion

This is the first study to demonstrate that the low-power laser irradiation of coronary artery during percutaneous coronary angioplasty (PCI) may have beneficial effect by limiting the local inflammation and thus the major factor of the restenosis cascade.

We have chosen the laser radiation with wavelength of 808 nm, taking into consideration the possibility to propagate the radiation into the lumen of the coronary arteries, their walls and surrounding tissues. Such wavelength is considered to have both anti-inflammatory effect as well as to stimulate endothelial regeneration. Up to date, there are a few experimental and clinical studies concerning the intravascular coronary laser illumination during angioplasty. The wavelength used in these procedures was initially set on 632 nm (Helium-neon laser) [11, 12] and then on 650 nm (semiconductor laser) [3, 4, 13, 14]. In another comparative studies, it has been shown that wavelength (780 nm) results in better healing of wounds as compared with shorter waves (670 nm) [15]; however, other authors mention greater efficiency of shorter wavelengths [16].

Serum level of IL 1β may initiate the process of restenosis after angioplasty procedure, probably by stimulating proliferation of smooth muscle cells [17]. The levels of IL 1β were higher in the control group at 6th and 12th hour following PCI, and then recovered to initial values within the 1st month following PCI. In laser-treated group, the radiation caused a decrease in concentration of IL 1β. This effect was maintained after 1 month. Other reports showed that the levels of interleukin increased [18], did not change [19] or decreased after radiation [20–22]. Furthermore, no differences in the levels of pro-inflammatory factors (IL 1β, IL 6 and IL 8, sICAM 1, sVCAM 1 in case of irradiation with *λ* = 904 nm [19] or *λ* = 632.8 nm [21]) were observed.

Similarly, IL 6 has also been demonstrated to stimulate the restenosis following PCI [23–25]. An increase in IL 6 level within several hours after the procedure was observed in both groups; however, lower values were observed in the irradiated group. After first month following PCI, a decrease in IL 6 level was observed, falling below initial values in irradiated group, while in the control group, the concentrations were slightly higher than the initial ones.

IL 10 inhibits inflammatory processes which may also result in decreased risk for restenosis [26, 27]. In our study, within the laser-treated group, we have not observed significant changes in IL 10 level within several hours following the procedure, when compared with initial values. Nevertheless, since a decrease in the level of IL 10 was observed in the control group, we may assume that the laser irradiation procedure prevented the decrease of anti-inflammatory interleukins.

A decrease in the lumen diameter greater than 50 % was the cutoff point for the diagnosis of restenosis, which is a commonly used threshold in clinical practice [7]. The loss of lumen following angioplasty procedure was more frequent and greater in magnitude in control group, as compared with the laser-treated group. Nevertheless, in subjects without restenosis, the lumen loss was similar in both groups. It is postulated that this phenomenon results from activation of numerous cytokins (e.g., IL-1 β [28]) and humoral response by the angioplasty procedure, which may play an important role in pathogenesis of restenosis. Laser irradiation may inhibit this process thus leading to minimizing the occurence of restenosis.

The investigated group was not numerous, but homogenous. In a vast majority of cases, the angioplasty procedure was followed by stent placement; therefore, the number of patients treated with only PCI was relatively low and the differences analyzed were statistically insignificant. Similarly, small number of cases with restenosis did not enable to perform statistical analysis of this process.

## Conclusions

Exposure of coronary artery to the intravascular low-energy laser irradiation results in increase of anti-inflammatory interleukins as well as in decrease of the pro-inflammatory interleukins levels. Inhibiting of inflammatory reaction may decrease the risk and magnitude of restenosis in patients undergoing coronary angioplasty.

## References

[1] Libby P, Simon DI, Rogers C, Topol E (2003). Inflammation and artery injury. Textbook of interventional cardiology.

[2] Casscells W, Roberts R, Towbin JA (1994). Mechanisms of restenosis. Texas Heart Inst J.

[3] Kaul U, Singh B, Sudan D, Ghose T, Kipshidze N (1998). Intravascular red light therapy after coronary stenting—angiographic and clinical follow-up study in humans. J Invas Cardiol.

[4] De Scheerder IK, Wang K, Nikolaychik V, Kaul U, Singh B, Sahota H, Keelan MH, Kipshidze N (2000). Long-term follow-up after coronary stenting and intravascular red laser therapy. Am J Cardiol.

[5] Derkacz A, Protasiewicz M, Poreba R, Szuba A, Andrzejak R (2010). Usefulness of intravascular low power laser illumination in preventing restenosis after percutaneous coronary intervention. Am J Cardiol.

[6] Holmes DR, Vlietstra R, Smith H, Vetrovec GW, Kent KM, Cowley MJ, Faxon DP, Gruentzig AR, Kelsey SF, Detre KM (1984). Restenosis after percutaneous transluminal coronary angioplasty (PTCA); report from the PTCA Registry of the National Heart, Lung and Blood Institute. Am J Cardiol.

[7] Chan A, Moliterno D, Topol E (2003). Restenosis. Textbook of interventional cardiology.

[8] Pawlik E, Grobelny A, Palasz Z, Abramski K, Derkacz A, Biały D, Protasiewicz M (2001). Method of intravascular low power laser illumination. Opt Appl.

[9] Derkacz A, Bialy D, Protasiewicz M, Beres-Pawlik E, Abramski K (2004). Intravascular low power laser light illumination—a new method in restenosis prevention. Proc SPIE.

[10] Derkacz A, Bialy D, Protasiewicz M, Pawlik E, Abramski K, Grobelny A, Pałasz Z, Nowosad H (2003). Photostimulation of coronary arteries with low power laser radiation: preliminary results for a new method in invasive cardiology therapy. Med Sci Monit.

[11] Kipshidze N, Keelan M, Nikolaychik V (1996) Impact of red light on restenosis. In: Waksman R, King S, Crocker I, Mould R (eds) Vascular brachytherapy. Nucleotron 165–175

[12] Kipshidze N, Sahota H, Komorowski R, Nikolaychik V, Keelan MH (1998). Photoremodeling of arterial wall reduces restenosis after balloon angioplasty in an atherosclerotic rabbit model. J Am Coll Cardiol.

[13] De Scheerder IK, Wang K, Zhou XR, Verbeken E, Keelan MH, Horn JB, Sahota H, Kipshidze N (1998). Intravascular low power red laser light as an adjunct to coronary stent implantation evaluated in a porcine coronary model. J Invas Cardiol.

[14] De Scheerder IK, Wang K, Kaul U, Singh B, Sahota H, Keelan MH, Kipshidze NN, Moses J (2001). Intravascular low-power laser irradiation after coronary stenting: long-term follow-up. Lasers Surg Med.

[15] Almeida-Lopes L, Rigau L, Zangaro RA, Guidugli-Neto J, Jaeger MM (2001). Comparison of low level laser therapy effects on cultured human gingival fibroblast proliferation using different irradiance and same fluence. Lasers Surg Med.

[16] Evans DH, Abrahamse H (2008). Efficacy of three different laser wavelengths for in vitro wound healing. Photodermatol Photoimmunol Photomed.

[17] Oemar B (1999). Is interleukin-1 beta a triggering factor for restenosis?. Cardiovasc Res.

[18] Yu HS, Chang KL, Yu CL, Chen JW, Chen GS (1996). Low-energy helium-neon laser irradiation stimulates interleukin-1 alpha and interleukin-8 release from cultured human keratinocytes. J Invest Dermatol.

[19] Bouma MG, Buurman WA, van den Wildenberg FA (1996). Low energy laser irradiation fails to modulate the inflammatory function of human monocytes and endothelial cells. Lasers Surg Med.

[20] Nomura K, Yamaguchi M, Abiko Y (2001). Inhibition of interleukin-1 beta production and gene expression in human gingival fibroblasts by low-energy laser irradiation. Lasers Med Sci.

[21] Safavi SM, Kazemi B, Esmaeili M, Fallah A, Modarresi A, Mir M (2008). Effects of low-level He–Ne laser irradiation on the gene expression of IL-1beta, TNF-alpha, IFN-gamma, TGF-beta, bFGF, and PDGF in rat’s gingiva. Lasers Med Sci.

[22] Yamaura M, Yao M, Yaroslavsky I, Cohen R, Smotrich M, Kochevar IE (2009). Low level light effects on inflammatory cytokine production by rheumatoid arthritis synoviocytes. Lasers Surg Med.

[23] Hojo Y, Ikeda U, Katsuki T, Mizuno O, Fukazawa H, Kurosaki K, Fujikawa H, Shimada K (2000). Interleukin 6 expression in coronary circulation after coronary angioplasty as risk factor for restenosis. Heart.

[24] Szkodzinski J, Blazelonis A, Wilczek K, Hudzik B, Romanowski W, Gasior M, Wojnar R, Lekston A, Polonski L, Zubelewicz-Szkodzinska B (2009). The role of interleukin-6 and trans forming growth factor-β1 in predicting restenosis within stented infarct-related artery. Int J Immunopathol Pharmacol.

[25] Ikeda U, Ito T, Shimada K (2001). Interleukin-6 and acute coronary syndrome. Clinic Cardiol.

[26] Feldman LJ, Aguirre L, Ziol M, Bridou JP, Nevo N, Michel JB, Steg PG (2000). Interleukin-10 inhibits hyperplasia after angioplasty or stent implantation in hypercholesterolemic rabbits. Circulation.

[27] Frangogiannis NG, Smith CW, Entman ML (2002). The inflammatory response in myocardial infarction. Cardiovasc Res.

[28] Pietersma A, Koffland M, de Wit E, Stijnen T, Koster J, Serruys P, Sluiter W (1995). Late lumen loss after coronary angioplasty is associated with the activation status of circulating phagocytes before treatment. Circulation.

